# Supplementary material for: Positive and negative incentive contrasts lead to relative value perception in ants
Source: eLife. 2019 Jul 2;8:e45450. doi: 10.7554/eLife.45450 (PMC6606023; doi:10.7554/eLife.45450)
Supplement: Figure 3—source data 1. [file elife-45450-fig3-data1.docx]

### GLMM Output for Reference Molarity as a continuous variable for the test visit (3)

Generalized linear mixed model fit by maximum likelihood (Laplace Approximation) ['glmerMod']

Family: poisson ( log )

Formula: Visit3ReturntoNestFrom0.5M ~ treatment + diff + I(diff^2) + (1 | colony/AntID)

Data: alldat

Control: glmerControl(optCtrl = list(maxfun = 2e+05))

AIC BIC logLik deviance df.resid

4775.8 4804.1 -2381.9 4763.8 820

Scaled residuals:

Min 1Q Median 3Q Max

-1.33666 -0.66148 0.00361 0.19325 1.32474

Random effects:

Groups Name Variance Std.Dev.

AntID:colony (Intercept) 1.02658 1.013

colony (Intercept) 0.05663 0.238

Number of obs: 826, groups: AntID:colony, 826; colony, 8

Fixed effects:

Estimate Std. Error z value Pr(>|z|)

(Intercept) 2.105142 0.112693 18.680 < 2e-16 ***

treatment -0.823320 0.084460 -9.748 < 2e-16 ***

diff 0.160300 0.010024 15.992 < 2e-16 ***

I(diff^2) -0.007430 0.001073 -6.927 4.29e-12 ***

---

Signif. codes: 0 ‘***’ 0.001 ‘**’ 0.01 ‘*’ 0.05 ‘.’ 0.1 ‘ ’ 1

Correlation of Fixed Effects:

(Intr) trtmnt diff

treatment -0.480

diff 0.085 -0.047

I(diff^2) -0.208 -0.029 -0.582

convergence code: 0

### Pairwise Comparisons Table

Table S2: Estimates, z-values and p-values for the pairwise comparisons of all twelve treatments on the third visit for inbound pheromone depositions on a 20 cm track right behind the food source of experiment 1. Pairwise comparisons were calculated in R with the lsmeans function and a Benjamini-Hochberg correction.

| **treatment** | **value** | **0.1** | **0.2** | **0.3** | **0.4** | **0.5** | **0.6** | **0.7** | **0.8** | **0.9** | **1** | **1.5** | **2** |
| --- | --- | --- | --- | --- | --- | --- | --- | --- | --- | --- | --- | --- | --- |
| **0.1** | Estimate |  | -0.42 | 0.04 | 0.30 | -0.08 | 0.05 | 0.12 | 0.18 | 0.14 | 0.94 | 0.94 | 1.34 |
|  | z-value |  | -2.23 | 0.21 | 1.44 | -0.41 | 0.25 | 0.60 | 0.87 | 0.70 | 4.11 | 4.41 | 6.07 |
|  | p-value |  | 1 | 1 | 1 | 1 | 1 | 1 | 1 | 1 | **<0.01** | **<.001** | **<.001** |
| **0.2** | Estimate | -0.42 |  | 0.47 | 0.72 | -0.51 | 0.48 | 0.55 | 0.60 | 0.57 | 1.37 | 1.36 | 1.77 |
|  | z-value | -2.23 |  | 2.63 | 3.83 | -2.86 | 2.56 | 3.00 | 3.22 | 3.11 | 6.44 | 6.98 | 8.62 |
|  | p-value | 1 |  | 0.56 | **<0.01** | 0.28 | 0.70 | 0.18 | 0.08 | 0.12 | **<.001** | **<.001** | **<.001** |
| **0.3** | Estimate | 0.04 | 0.47 |  | 0.26 | -0.04 | 0.01 | 0.08 | 0.14 | 0.10 | 0.64 | 0.64 | 1.04 |
|  | z-value | 0.21 | 2.63 |  | 1.33 | -0.22 | 0.06 | 0.43 | 0.71 | 0.54 | 2.86 | 3.03 | 4.75 |
|  | p-value | 1 | 0.56 |  | 1 | 1 | 1 | 1 | 1 | 1 | 0.28 | 0.16 | **<.001** |
| **0.4** | Estimate | 0.30 | 0.72 | 0.26 |  | 0.22 | -0.25 | -0.18 | -0.12 | -0.16 | 0.64 | 0.64 | 1.04 |
|  | z-value | 1.44 | 3.83 | 1.33 |  | 1.12 | -1.23 | -0.89 | -0.59 | -0.79 | 2.86 | 3.03 | 4.75 |
|  | p-value | 1 | **<0.01** | 1 |  | 1 | 1 | 1 | 1 | 1 | 0.28 | 0.16 | **<.001** |
| **0.5** | Estimate | -0.08 | -0.51 | -0.04 | 0.22 |  | -0.03 | 0.04 | 0.10 | 0.06 | 0.86 | 0.86 | 1.26 |
|  | z-value | -0.41 | -2.86 | -0.22 | 1.12 |  | -0.15 | 0.22 | 0.50 | 0.32 | 3.97 | 4.28 | 6.03 |
|  | p-value | 1 | 0.28 | 1 | 1 |  | 1 | 1 | 1 | 1 | **<0.01** | **<0.01** | **<.001** |
| **0.6** | Estimate | 0.05 | 0.48 | 0.01 | -0.25 | -0.03 |  | 0.07 | 0.12 | 0.09 | 0.89 | 0.88 | 1.29 |
|  | z-value | 0.25 | 2.56 | 0.06 | -1.23 | -0.15 |  | 0.36 | 0.63 | 0.45 | 3.97 | 4.25 | 5.94 |
|  | p-value | 1 | 0.70 | 1 | 1 | 1 |  | 1 | 1 | 1 | **<0.01** | **<0.01** | **<.001** |
| **0.7** | Estimate | 0.12 | 0.55 | 0.08 | -0.18 | 0.04 | 0.07 |  | 0.05 | 0.02 | 0.82 | 0.81 | 1.22 |
|  | z-value | 0.60 | 3.00 | 0.43 | -0.89 | 0.22 | 0.36 |  | 0.28 | 0.10 | 3.71 | 3.98 | 5.70 |
|  | p-value | 1 | 0.18 | 1 | 1 | 1 | 1 |  | 1 | 1 | **<0.05** | **<0.01** | **<.001** |
| **0.8** | Estimate | 0.18 | 0.60 | 0.14 | -0.12 | 0.10 | 0.12 | 0.05 |  | -0.04 | 0.76 | 0.76 | 1.16 |
|  | z-value | 0.87 | 3.22 | 0.71 | -0.59 | 0.50 | 0.63 | 0.28 |  | -0.18 | 3.39 | 3.64 | 5.35 |
|  | p-value | 1 | 0.08 | 1 | 1 | 1 | 1 | 1 |  | 1 | **<0.05** | **<0.05** | **<.001** |
| **0.9** | Estimate | 0.14 | 0.57 | 0.10 | -0.16 | 0.06 | 0.09 | 0.02 | -0.04 |  | 0.80 | 0.80 | 1.20 |
|  | z-value | 0.70 | 3.11 | 0.54 | -0.79 | 0.32 | 0.45 | 0.10 | -0.18 |  | 3.62 | 3.89 | 5.62 |
|  | p-value | 1 | 0.12 | 1 | 1 | 1 | 1 | 1 | 1 |  | **<0.05** | **<0.01** | **<.001** |
| **1** | Estimate | 0.94 | 1.37 | 0.64 | 0.64 | 0.86 | 0.89 | 0.82 | 0.76 | 0.80 |  | -0.01 | 0.40 |
|  | z-value | 4.11 | 6.44 | 2.86 | 2.86 | 3.97 | 3.97 | 3.71 | 3.39 | 3.62 |  | -0.02 | 1.67 |
|  | p-value | **<0.01** | **<.001** | 0.28 | 0.28 | **<0.01** | **<0.01** | **<0.05** | **<0.05** | **<0.05** |  | 1 | 1 |
| **1.5** | Estimate | 0.94 | 1.36 | 0.64 | 0.64 | 0.86 | 0.88 | 0.81 | 0.76 | 0.80 | -0.01 |  | 0.40 |
|  | z-value | 4.41 | 6.98 | 3.03 | 3.03 | 4.28 | 4.25 | 3.98 | 3.64 | 3.89 | -0.02 |  | 1.81 |
|  | p-value | **<.001** | **<.001** | 0.16 | 0.16 | **<0.01** | **<0.01** | **<0.01** | **<0.05** | **<0.01** | 1 |  | 1 |
| **2** | Estimate | 1.34 | 1.77 | 1.04 | 1.04 | 1.26 | 1.29 | 1.22 | 1.16 | 1.20 | 0.40 | 0.40 |  |
|  | z-value | 6.07 | 8.62 | 4.75 | 4.75 | 6.03 | 5.94 | 5.70 | 5.35 | 5.62 | 1.67 | 1.81 |  |
|  | p-value | **<.001** | **<.001** | **<.001** | **<.001** | **<.001** | **<.001** | **<.001** | **<.001** | **<.001** | 1 | 1 |  |

### GLMM Output for Reference Molarity as a continuous variable for the training visits (1 & 2)

Generalized linear mixed model fit by maximum likelihood (Laplace Approximation) ['glmerMod']

Family: poisson ( log )

Formula: PheroDepoNest ~ treatment + visit + (1 | colony/AntID)

Data: pherodata1n2

Control: glmerControl(optCtrl = list(maxfun = 10000))

AIC BIC logLik deviance df.resid

9858.7 9885.7 -4924.3 9848.7 1623

Scaled residuals:

Min 1Q Median 3Q Max

-1.2900 -0.7674 0.0243 0.1824 0.5008

Random effects:

Groups Name Variance Std.Dev.

AntID:colony (Intercept) 1.4729 1.2136

colony (Intercept) 0.1128 0.3358

Number of obs: 1628, groups: AntID:colony, 1628; colony, 8

Fixed effects:

Estimate Std. Error z value Pr(>|z|)

(Intercept) 0.47214 0.13866 3.405 0.000661 ***

treatment 0.86247 0.06220 13.867 < 2e-16 ***

visitvisit2 0.31065 0.06686 4.646 3.38e-06 ***

---

Signif. codes: 0 ‘***’ 0.001 ‘**’ 0.01 ‘*’ 0.05 ‘.’ 0.1 ‘ ’ 1

Correlation of Fixed Effects:

(Intr) trtmnt

treatment -0.370

visitvisit2 -0.260 0.016
