# Supplementary material for: Positive and negative incentive contrasts lead to relative value perception in ants
Source: eLife. 2019 Jul 2;8:e45450. doi: 10.7554/eLife.45450 (PMC6606023; doi:10.7554/eLife.45450)
Supplement: Figure 4—source data 3. [file elife-45450-fig4-data3.docx]

### GLMM Output Training visits (1-8)

Generalized linear mixed model fit by maximum likelihood (Laplace Approximation) ['glmerMod']

Family: Negative Binomial(1.7757) ( log )

Formula: PheroDepositionsOutbound ~ HighLowMolarityscent * scale(visit) +

Scent.Molarity + (1 | Colony/AntID)

Data: visit1to8PDfood

Control: glmerControl(optCtrl = list(maxfun = 10000))

AIC BIC logLik deviance df.resid

2823.6 2857.3 -1403.8 2807.6 489

Scaled residuals:

Min 1Q Median 3Q Max

-1.2461 -0.7847 -0.1812 0.4848 3.0833

Random effects:

Groups Name Variance Std.Dev.

AntID:Colony (Intercept) 0.3391 0.5823

Colony (Intercept) 0.1834 0.4283

Number of obs: 497, groups: AntID:Colony, 72; Colony, 6

Fixed effects:

Estimate Std. Error z value Pr(>|z|)

(Intercept) 1.44392 0.20108 7.181 6.92e-13 ***

HighLowMolarityscentLow 0.23718 0.08200 2.892 0.00382 **

scale(visit) 0.75973 0.05946 12.778 < 2e-16 ***

Scent.MolarityRosemary -0.04930 0.08121 -0.607 0.54380

HighLowMolarityscentLow:scale(visit) -0.93987 0.09398 -10.001 < 2e-16 ***

---

Signif. codes: 0 ‘***’ 0.001 ‘**’ 0.01 ‘*’ 0.05 ‘.’ 0.1 ‘ ’ 1

Correlation of Fixed Effects:

(Intr) HghLML scl(v) Scn.MR

HghLwMlrtyL -0.171

scale(vist) -0.064 0.134

Scnt.MlrtyR -0.183 -0.073 0.011

HghLwMlL:() 0.042 -0.059 -0.628 -0.006
