# Supplementary material for: Positive and negative incentive contrasts lead to relative value perception in ants
Source: eLife. 2019 Jul 2;8:e45450. doi: 10.7554/eLife.45450 (PMC6606023; doi:10.7554/eLife.45450)
Supplement: Figure 4—source data 4. [file elife-45450-fig4-data4.docx]

### GLMM Output Test visit (9)

Generalized linear mixed model fit by maximum likelihood (Laplace Approximation) ['glmerMod']

Family: poisson ( log )

Formula: PheroDepositiontoFoodSource ~ HighLowMolarityscent + Scent.Molarity + (1 | Colony/AntID)

Data: visit9PDfood

AIC BIC logLik deviance df.resid

470.1 481.2 -230.0 460.1 63

Scaled residuals:

Min 1Q Median 3Q Max

-2.2986 -1.5570 -0.2855 1.1586 3.7727

Random effects:

Groups Name Variance Std.Dev.

AntID:Colony (Intercept) 0.02709 0.1646

Colony (Intercept) 0.09001 0.3000

Number of obs: 68, groups: AntID:Colony, 26; Colony, 6

Fixed effects:

Estimate Std. Error z value Pr(>|z|)

(Intercept) 2.58102 0.14385 17.942 <2e-16 ***

HighLowMolarityscentLow -1.31679 0.10172 -12.945 <2e-16 ***

Scent.MolarityRosemary 0.04149 0.09189 0.452 0.652

---

Signif. codes: 0 ‘***’ 0.001 ‘**’ 0.01 ‘*’ 0.05 ‘.’ 0.1 ‘ ’ 1

Correlation of Fixed Effects:

(Intr) HghLML

HghLwMlrtyL -0.152

Scnt.MlrtyR -0.302 -0.005
