# Supplementary material for: Positive and negative incentive contrasts lead to relative value perception in ants
Source: eLife. 2019 Jul 2;8:e45450. doi: 10.7554/eLife.45450 (PMC6606023; doi:10.7554/eLife.45450)
Supplement: Figure 4—source data 5. [file elife-45450-fig4-data5.docx]

### GLMM Output Training visits (1-8)

Generalized linear mixed model fit by maximum likelihood (Laplace Approximation) ['glmerMod']

Family: Negative Binomial(1.2723) ( log )

Formula: PheroDepositiontoNest ~ HighLowMolarityscent * scale(visit) +

Scent.Molarity + (1 | Colony/AntID)

Data: visit1to8PD

AIC BIC logLik deviance df.resid

2328.6 2363.2 -1156.3 2312.6 548

Scaled residuals:

Min 1Q Median 3Q Max

-1.0489 -0.6258 -0.3777 0.3305 4.3407

Random effects:

Groups Name Variance Std.Dev.

AntID:Colony (Intercept) 0.5662 0.7525

Colony (Intercept) 0.1077 0.3282

Number of obs: 556, groups: AntID:Colony, 72; Colony, 6

Fixed effects:

Estimate Std. Error z value Pr(>|z|)

(Intercept) 1.88318 0.18074 10.419 < 2e-16 ***

HighLowMolarityscentLow -2.49647 0.12831 -19.456 < 2e-16 ***

scale(visit) -0.03965 0.06449 -0.615 0.538708

Scent.MolarityRosemary -0.38935 0.11274 -3.453 0.000554 ***

HighLowMolarityscentLow:scale(visit) -0.53181 0.12047 -4.414 1.01e-05 ***

---

Signif. codes: 0 ‘***’ 0.001 ‘**’ 0.01 ‘*’ 0.05 ‘.’ 0.1 ‘ ’ 1

Correlation of Fixed Effects:

(Intr) HghLML scl(v) Scn.MR

HghLwMlrtyL -0.166

scale(vist) -0.065 0.102

Scnt.MlrtyR -0.263 0.074 0.003

HghLwMlL:() 0.029 0.284 -0.525 0.039
