# Supplementary material for: Positive and negative incentive contrasts lead to relative value perception in ants
Source: eLife. 2019 Jul 2;8:e45450. doi: 10.7554/eLife.45450 (PMC6606023; doi:10.7554/eLife.45450)
Supplement: Figure 4—source data 6. [file elife-45450-fig4-data6.docx]

### GLMM Output Test visit (9)

Generalized linear mixed model fit by maximum likelihood (Laplace Approximation) ['glmerMod']

Family: poisson ( log )

Formula: PheroDepositiontoNest ~ HighLowMolarityscent + Scent.Molarity + (1 | Colony/AntID)

Data: visit9PD

Control: glmerControl(optCtrl = list(maxfun = 10000))

AIC BIC logLik deviance df.resid

266.2 277.4 -128.1 256.2 65

Scaled residuals:

Min 1Q Median 3Q Max

-1.7997 -0.6496 -0.4797 0.3529 2.6454

Random effects:

Groups Name Variance Std.Dev.

AntID:Colony (Intercept) 1.6094 1.2686

Colony (Intercept) 0.2874 0.5361

Number of obs: 70, groups: AntID:Colony, 43; Colony, 6

Fixed effects:

Estimate Std. Error z value Pr(>|z|)

(Intercept) 0.5361 0.4185 1.281 0.20013

HighLowMolarityscentLow -1.6521 0.5460 -3.026 0.00248 **

Scent.MolarityRosemary -0.1982 0.2861 -0.693 0.48853

---

Signif. codes: 0 ‘***’ 0.001 ‘**’ 0.01 ‘*’ 0.05 ‘.’ 0.1 ‘ ’ 1

Correlation of Fixed Effects:

(Intr) HghLML

HghLwMlrtyL -0.363

Scnt.MlrtyR -0.322 -0.055
