# Supplementary material for: Positive and negative incentive contrasts lead to relative value perception in ants
Source: eLife. 2019 Jul 2;8:e45450. doi: 10.7554/eLife.45450 (PMC6606023; doi:10.7554/eLife.45450)
Supplement: Figure 4—figure supplement 1—source data 1. [file elife-45450-fig4-figsupp1-data1.docx]

## Analysis of drinking interruption behaviour

Generalized linear mixed model fit by maximum likelihood (Laplace

Approximation) [glmerMod]

Family: poisson ( log )

Formula: Pauses ~ HighLow + Scent + (1 | Colony)

Random effects:

Groups Name Variance Std.Dev.

Colony (Intercept) 4.975e-17 7.053e-09

Number of obs: 68, groups: Colony, 6

Fixed effects:

Estimate Std. Error z value Pr(>|z|)

(Intercept) 1.3896 0.1055 13.167 < 2e-16 ***

HighLowLow -0.3551 0.1295 -2.741 0.00613 **

ScentRosemary 0.1520 0.1273 1.194 0.23248

---

## Signif. codes: 0 '***' 0.001 '**' 0.01 '*' 0.05 '.' 0.1 ' ' 1

##

## Correlation of Fixed Effects:

## (Intr) HghLwL

## HighLowLow -0.391

## ScentRosmry -0.665 -0.095
