# Supplementary material for: Positive and negative incentive contrasts lead to relative value perception in ants
Source: eLife. 2019 Jul 2;8:e45450. doi: 10.7554/eLife.45450 (PMC6606023; doi:10.7554/eLife.45450)
Supplement: Supplementary file 1. [file elife-45450-supp1.docx]

| **Experiment** | **Reference /Ex- pected Molarity** | **Sample Size** | **Nr. of Ants excluded** | **proportion finished [%]** | **Mean Food Acceptance** | **Median Pheromone Depositions to Nest [20cm]** | **Median Pheromone Depositions to Food [20cm]** |
| --- | --- | --- | --- | --- | --- | --- | --- |
| **Relative Value Perception**  **(Experiment 1)** | **0.1M** | 57 | 0 | 100 | 0.94 | 8 | 2 |
|  | **0.2M** | 80 | 0 | 100 | 0.83 | 10 | 8 |
|  | **0.3M** | 76 | 2 | 97.43 | 0.76 | 6.5 | 6 |
|  | **0.4M** | 66 | 3 | 95.65 | 0.73 | 5 | 6 |
|  | **0.5M** | 77 | 2 | 97.47 | 0.71 | 7 | 10 |
|  | **0.6M** | 65 | 1 | 98.48 | 0.67 | 7 | 9 |
|  | **0.7M** | 73 | 2 | 97.33 | 0.62 | 5 | 11 |
|  | **0.8M** | 66 | 2 | 97.06 | 0.61 | 7 | 10 |
|  | **0.9M** | 72 | 1 | 98.63 | 0.60 | 6 | 12 |
|  | **1M** | 55 | 5 | 91.66 | 0.58 | 2 | 10 |
|  | **1.5M** | 72 | 7 | 91.14 | 0.42 | 1 | 13 |
|  | **2M** | 70 | 0 | 100 | 0.39 | 0 | 14 |
| **Scent Training Control**  **(Experiment 2)** | **1.5M** | 38 | 2 | 95 | 0.70 | 0 | 15 |
|  | **0.25M** | 32 | 0 | 100 | 0.83 | 0 | 2 |
| **Trophallaxis (Experiment 3)** | **0.16M** | 63 | 0 | 100 | 0.86 |  |  |
|  | **0.5M** | 52 | 0 | 100 | 0.76 |  |  |
|  | **1.5M** | 53 | 0 | 100 | 0.58 |  |  |
