# Supplementary material for: Positive and negative incentive contrasts lead to relative value perception in ants
Source: eLife. 2019 Jul 2;8:e45450. doi: 10.7554/eLife.45450 (PMC6606023; doi:10.7554/eLife.45450)
Supplement: Supplementary file 12 [file elife-45450-fig2-data1.docx]

### CLMM Output for Reference Molarity as a continuous variable for the test visit (3)

## Cumulative Link Mixed Model fitted with the Laplace approximation
## 
## formula: FoodAcceptance3rdvisit ~ ReferenceMolarity + (1 | colony)
## data:    thirdvisit
## 
##  link  threshold nobs logLik  AIC     niter    max.grad cond.H 
##  logit flexible  829  -574.07 1156.14 143(305) 1.81e-05 1.7e+01
## 
## Random effects:
##  Groups Name        Variance Std.Dev.

##  colony (Intercept) 0.2142   0.4628  
## Number of groups:  colony 8 
## 
## Coefficients:
##           Estimate Std. Error z value Pr(>|z|)    
## treatment  -2.5908     0.1909  -13.57   <2e-16 ***
## ---
## Signif. codes:  0 '***' 0.001 '**' 0.01 '*' 0.05 '.' 0.1 ' ' 1

### Pairwise Comparisons Table

Table S1: Estimates, z-values and p-values for the pairwise comparisons of all twelve treatments on the third visit for food acceptance of experiment 1. Pairwise comparisons were calculated in R with the lsmeans function and a Benjamini-Hochberg correction.

| treatment | value | 0.1 | 0.2 | 0.3 | 0.4 | 0.5 | 0.6 | 0.7 | 0.8 | 0.9 | 1 | 1.5 | 2 |
| --- | --- | --- | --- | --- | --- | --- | --- | --- | --- | --- | --- | --- | --- |
| 0.1 | Estimate |  | 1.35 | 1.98 | 2.11 | -2.39 | 2.69 | 2.98 | 3.18 | 3.34 | 3.43 | 5.19 | 5.63 |
| z-value |  | 0.48 | 0.47 | 0.48 | -5.05 | 0.49 | 0.49 | 0.50 | 0.49 | 0.52 | 0.53 | 0.53 |
| p-value |  | 0.31 | <0.01 | <.001 | <.001 | <.001 | <.001 | <.001 | <.001 | <.001 | <.001 | <.001 |
| 0.2 | Estimate | 1.35 |  | 0.63 | 0.75 | -1.04 | 1.34 | 1.63 | 1.83 | 1.99 | 2.08 | 3.84 | 4.27 |
| z-value | 0.48 |  | 1.87 | 2.17 | 0.33 | 3.73 | 4.61 | 4.98 | 5.55 | 5.24 | 9.32 | 10.30 |
| p-value | 0.31 |  | 1 | 1 | 0.13 | <0.05 | <.001 | <.001 | <.001 | <.001 | <.001 | <.001 |
| 0.3 | Estimate | 1.98 | 0.63 |  | 0.12 | -0.41 | 0.70 | 1.00 | 1.20 | 1.36 | 1.45 | 3.21 | 3.64 |
| z-value | 0.47 | 1.87 |  | 0.36 | 0.33 | 1.99 | 2.87 | 3.32 | 3.85 | 3.69 | 7.90 | 8.91 |
| p-value | <0.01 | 1 |  | 1 | 1 | 1 | 0.27 | 0.059 | <0.01 | <0.05 | <.001 | <.001 |
| 0.4 | Estimate | 2.11 | 0.75 | 0.12 |  | -0.28 | 0.58 | 0.88 | 1.08 | 1.23 | 1.33 | 3.09 | 3.52 |
| z-value | 0.48 | 2.17 | 0.36 |  | 0.34 | 1.59 | 2.43 | 2.87 | 3.37 | 3.30 | 7.40 | 8.38 |
| p-value | <.001 | 1 | 1 |  | 1 | 1 | 0.99 | 0.27 | <0.05 | 0.06 | <.001 | <.001 |
| 0.5 | Estimate | -2.39 | -1.04 | -0.41 | -0.28 |  | 0.30 | 0.59 | 0.80 | 0.95 | 1.04 | 2.80 | 3.24 |
| z-value | -5.05 | 0.33 | 0.33 | 0.34 |  | 0.35 | 0.35 | 0.36 | 0.35 | 0.39 | 0.40 | 0.41 |
| p-value | <.001 | 0.13 | 1 | 1 |  | 1 | 1 | 1 | 0.44 | 0.5 | <.001 | <.001 |
| 0.6 | Estimate | 2.69 | 1.34 | 0.70 | 0.58 | 0.30 |  | 0.30 | 0.50 | 0.65 | 0.74 | 2.50 | 2.94 |
| z-value | 0.49 | 3.73 | 1.99 | 1.59 | 0.35 |  | 0.80 | 1.31 | 1.76 | 1.82 | 5.97 | 6.98 |
| p-value | <.001 | <0.05 | 1 | 1 | 1 |  | 1 | 1 | 1 | 1 | <.001 | <.001 |
| 0.7 | Estimate | 2.98 | 1.63 | 1.00 | 0.88 | 0.59 | 0.30 |  | 0.20 | 0.35 | 0.45 | 2.21 | 2.64 |
| z-value | 0.49 | 4.61 | 2.87 | 2.43 | 0.35 | 0.80 |  | 0.53 | 0.97 | 1.10 | 5.36 | 6.38 |
| p-value | <.001 | <.001 | 0.27 | 0.99 | 1 | 1 |  | 1 | 1 | 1 | <.001 | <.001 |
| 0.8 | Estimate | 3.18 | 1.83 | 1.2 | 1.08 | 0.80 | 0.50 | 0.20 |  | 0.15 | 0.25 | 2.00 | 2.44 |
| z-value | 0.50 | 4.98 | 3.32 | 2.87 | 0.36 | 1.31 | 0.53 |  | 0.41 | 0.59 | 4.77 | 5.79 |
| p-value | <.001 | <.001 | 0.059 | 0.27 | 1 | 1 | 1 |  | 1 | 1 | <.001 | <.001 |
| 0.9 | Estimate | 3.34 | 1.99 | 1.36 | 1.23 | 0.95 | 0.65 | 0.35 | 0.15 |  | 0.09 | 1.85 | 2.28 |
| z-value | 0.49 | 5.55 | 3.85 | 3.37 | 0.35 | 1.76 | 0.97 | 0.41 |  | 0.23 | 4.53 | 5.58 |
| p-value | <.001 | <.001 | <0.01 | <0.05 | 0.44 | 1 | 1 | 1 |  | 1 | <.001 | <.001 |
| 1 | Estimate | 3.43 | 2.08 | 1.45 | 1.33 | 1.04 | 0.74 | 0.45 | 0.25 | 0.09 |  | 1.76 | 2.19 |
| z-value | 0.52 | 5.24 | 3.69 | 3.30 | 0.39 | 1.82 | 1.10 | 0.59 | 0.23 |  | 3.99 | 4.97 |
| p-value | <.001 | <.001 | <0.05 | 0.06 | 0.5 | 1 | 1 | 1 | 1 |  | <0.05 | <.001 |
| 1.5 | Estimate | 5.19 | 3.84 | 3.21 | 3.09 | 2.8 | 2.50 | 2.21 | 2.00 | 1.85 | 1.76 |  | 0.43 |
| z-value | 0.53 | 9.32 | 7.90 | 7.40 | 0.40 | 5.97 | 5.36 | 4.77 | 4.53 | 3.99 |  | 1.14 |
| p-value | <.001 | <.001 | <.001 | <.001 | <.001 | <.001 | <.001 | <.001 | <.001 | <0.05 |  | 1 |
| 2 | Estimate | 5.63 | 4.27 | 3.64 | 3.52 | 3.24 | 2.94 | 2.64 | 2.44 | 2.28 | 2.19 | 0.43 |  |
| z-value | 0.53 | 10.30 | 8.91 | 8.38 | 0.41 | 6.98 | 6.38 | 5.79 | 5.58 | 4.97 | 1.14 |  |
| p-value | <.001 | <.001 | <.001 | <.001 | <.001 | <.001 | <.001 | <.001 | <.001 | <.001 | 1 |  |

### CLMM Output for Reference Molarity as a continuous variable for the training visits (1 & 2)

Cumulative Link Mixed Model fitted with the Laplace approximation

formula: FoodAcceptance ~ treatment + visit + (1 | colony/AntID)

data:    foodacc1n2

 link  threshold nobs logLik   AIC     niter     max.grad cond.H 

 logit flexible  1639 -1136.34 2284.69 315(1264) 7.39e-04 1.3e+01

Random effects:

 Groups       Name        Variance Std.Dev.

 AntID:colony (Intercept) 2.4944   1.5794  

 colony       (Intercept) 0.2064   0.4543  

Number of groups:  AntID:colony 833,  colony 8 

Coefficients :          
  Estimate Std. Error z value Pr(>|z|)    

treatment     1.9734     0.2046   9.646  < 2e-16 ***

visitvisit1   0.6039     0.1268   4.764  1.9e-06 ***

---

Signif. codes:  0 ‘***’ 0.001 ‘**’ 0.01 ‘*’ 0.05 ‘.’ 0.1 ‘ ’ 1

Threshold coefficients:

      Estimate Std. Error z value

0|0.5  -3.4391     0.2966  -11.60

0.5|1   1.2999     0.2372    5.48
