# Supplementary material for: Positive and negative incentive contrasts lead to relative value perception in ants
Source: eLife. 2019 Jul 2;8:e45450. doi: 10.7554/eLife.45450 (PMC6606023; doi:10.7554/eLife.45450)
Supplement: Supplementary file 13 [file elife-45450-fig4-data1.docx]

### CLMM Output Training visits (1-8)

Cumulative Link Mixed Model fitted with the Laplace approximation

formula: FoodAcceptability ~ HighLowMolarityscent * scale(visit) + Scent.Molarity +      (1 | Colony/AntID)

data:    visit1to8FA

 link  threshold nobs logLik  AIC    niter     max.grad cond.H 

 logit flexible  569  -235.23 486.45 393(1182) 5.02e-06 2.9e+02

Random effects:

 Groups       Name        Variance Std.Dev.

 AntID:Colony (Intercept) 0.9769   0.9884  

 Colony       (Intercept) 0.1628   0.4035  

Number of groups:  AntID:Colony 72,  Colony 6 

Coefficients:

                                     Estimate Std. Error z value Pr(>|z|)    

HighLowMolarityscentLow               -7.3394     0.8224  -8.924  < 2e-16 ***

scale(visit)                           0.9140     0.7556   1.210 0.226412    

Scent.MolarityRosemary                 0.1106     0.3462   0.319 0.749418    

HighLowMolarityscentLow:scale(visit)  -2.8408     0.7828  -3.629 0.000284 ***

---

Signif. codes:  0 ‘***’ 0.001 ‘**’ 0.01 ‘*’ 0.05 ‘.’ 0.1 ‘ ’ 1

Threshold coefficients:

      Estimate Std. Error z value

0|0.5  -8.9378     0.8942  -9.995

0.5|1  -5.1885     0.7713  -6.727
