# Supplementary material for: Positive and negative incentive contrasts lead to relative value perception in ants
Source: eLife. 2019 Jul 2;8:e45450. doi: 10.7554/eLife.45450 (PMC6606023; doi:10.7554/eLife.45450)
Supplement: Supplementary file 14 [file elife-45450-fig4-data2.docx]

### CLMM Output Test visit (9)

Cumulative Link Mixed Model fitted with the Laplace approximation

formula: FoodAcceptability ~ Scent.Molarity + HighLowMolarityscent +      (1 | Colony)

data:    visit9FA

 link  threshold nobs logLik AIC    niter    max.grad cond.H 

 logit flexible  70   -57.27 124.54 167(349) 6.49e-06 7.8e+01

Random effects:

 Groups Name        Variance Std.Dev.

 Colony (Intercept) 0.07835  0.2799  

Number of groups:  Colony 6 

Coefficients:

                        Estimate Std. Error z value Pr(>|z|)  

Scent.MolarityRosemary   -0.1698     0.5118  -0.332   0.7400  

HighLowMolarityscentLow   1.0430     0.5109   2.041   0.0412 *

---

Signif. codes:  0 ‘***’ 0.001 ‘**’ 0.01 ‘*’ 0.05 ‘.’ 0.1 ‘ ’ 1

Threshold coefficients:

      Estimate Std. Error z value

0|0.5 -2.55654    0.61920  -4.129

0.5|1  0.07686    0.42852   0.179
