# Supplementary material for: Positive and negative incentive contrasts lead to relative value perception in ants
Source: eLife. 2019 Jul 2;8:e45450. doi: 10.7554/eLife.45450 (PMC6606023; doi:10.7554/eLife.45450)
Supplement: Supplementary file 15 [file elife-45450-fig5-data1.docx]

## Food Acceptance 

### CLMM Output for Reference Molarity as a continuous variable

Cumulative Link Mixed Model fitted with the Laplace approximation

formula: FoodAcceptability ~ scale(ReferenceMolarity) * scale(TrophallaxisTime_seconds) +      (1 | Colony)

data:    alldat

 link  threshold nobs logLik  AIC    niter    max.grad cond.H 

 logit flexible  150  -112.45 236.90 304(915) 4.60e-05 1.3e+01

Random effects:

 Groups Name        Variance Std.Dev.

 Colony (Intercept) 0.3991   0.6317  

Number of groups:  Colony 8 

Coefficients:

                                                       Estimate Std. Error z value Pr(>|z|)    

scale(ReferenceMolarity)                                -0.5727     0.1865  -3.070 0.002138 ** 

scale(TrophallaxisTime_seconds)                         -0.7024     0.1939  -3.623 0.000292 ***

scale(ReferenceMolarity):scale(TrophallaxisTime_seconds)-0.4841     0.2080  -2.328 0.019933 *  

---

Signif. codes:  0 ‘***’ 0.001 ‘**’ 0.01 ‘*’ 0.05 ‘.’ 0.1 ‘ ’ 1

Threshold coefficients:

      Estimate Std. Error z value

0|0.5  -3.4829     0.5006  -6.957

0.5|1  -0.4237     0.3028  -1.399

### Pairwise Comparisons Table

Table S3: Estimates, z-values and p-values for the pairwise comparisons of all three treatments for the recruit for food acceptance of experiment 3. Pairwise comparisons were calculated in R with the lsmeans function and a Benjamini-Hochberg correction.

| treatment | value | 0.166 | 0.5 | 1.5 |
| --- | --- | --- | --- | --- |
| 0.166 | Estimate |  | 0.92 | 1.53 |
| z-value |  | 2.00 | 3.33 |
| p-value |  | 0.13 | <0.01 |
| 0.5 | Estimate | 0.92 |  | 0.61 |
| z-value | 2.00 |  | 1.29 |
| p-value | 0.13 |  | 0.59 |
| 1.5 | Estimate | 1.53 | 0.61 |  |
| z-value | 3.33 | 1.29 |  |
| p-value | <0.01 | 0.59 |  |

## Trophallaxis Time in seconds

### GLMM Output

## Generalized linear mixed model fit by maximum likelihood (Laplace Approximation) [glmerMod]
##  Family: poisson  ( log )
## Formula: TrophTime ~ ReferenceMolarity + (1 | Colony)
##    Data: alldat
## 
##      AIC      BIC   logLik deviance df.resid 
##   1790.1   1799.2   -892.1   1784.1      147 
## 
## Scaled residuals: 
##     Min      1Q  Median      3Q     Max 
## -4.9748 -2.0702 -0.4718  1.4715  7.5692 
## 
## Random effects:
##  Groups Name        Variance Std.Dev.
##  Colony (Intercept) 0.01304  0.1142  
## Number of obs: 150, groups:  Colony, 8
## 
## Fixed effects:
##                   Estimate Std. Error z value Pr(>|z|)    
## (Intercept)        3.30593    0.04702   70.31  < 2e-16 ***
## ReferenceMolarity  0.12719    0.02654    4.79 1.65e-06 ***
## ---
## Signif. codes:  0 '***' 0.001 '**' 0.01 '*' 0.05 '.' 0.1 ' ' 1

### Pairwise Comparisons Table

Table S4: Estimates, z-values and p-values for the pairwise comparisons of all three treatments for the recruit for trophallaxis time in seconds of experiment 3. Pairwise comparisons were calculated in R with the lsmeans function and a Benjamini-Hochberg correction.

| treatment | value | 0.166 | 0.5 | 1.5 |
| --- | --- | --- | --- | --- |
| 0.166 | Estimate |  | -0.12 | -0.19 |
| z-value |  | -3.08 | -5.08 |
| p-value |  | <0.01 | <.001 |
| 0.5 | Estimate | -0.12 |  | -0.07 |
| z-value | -3.08 |  | -1.76 |
| p-value | <0.01 |  | 0.23 |
| 1.5 | Estimate | -0.19 | -0.07 |  |
| z-value | -5.08 | -1.76 |  |
| p-value | <.001 | 0.23 |  |
